# *CCR5*-Mediated Signaling is Involved in Invasion of Glioblastoma Cells in Its Microenvironment

**DOI:** 10.3390/ijms21124199

**Published:** 2020-06-12

**Authors:** Metka Novak, Miha Koprivnikar Krajnc, Barbara Hrastar, Barbara Breznik, Bernarda Majc, Mateja Mlinar, Ana Rotter, Andrej Porčnik, Jernej Mlakar, Katja Stare, Richard G. Pestell, Tamara Lah Turnšek

**Affiliations:** 1 Department of Genetic Toxicology and Cancer Biology, National Institute of Biology, 1000 Ljubljana, Slovenia; metka.novak@nib.si (M.N.); mkoprivnikarkrajnc@gmail.com (M.K.K.); barbara.hrastar@gmail.com (B.H.); barbara.breznik@nib.si (B.B.); bernarda.majc@nib.si (B.M.); mateja.mlinar@nib.si (M.M.); ana.rotter@nib.si (A.R.); 2 Jožef Stefan International Postgraduate School, 1000 Ljubljana, Slovenia; 3 Department of Neurosurgery, University Medical Centre Ljubljana, 1000 Ljubljana, Slovenia; andrej.porcnik@ukclj.si; 4 Institute of Pathology, Faculty of Medicine, University of Ljubljana, 1000 Ljubljana, Slovenia; Jernej.MLAKAR@mf.uni-lj.si; 5 Department of Biotechnology and Systems Biology, National Institute of Biology, 1000 Ljubljana, Slovenia; katja.stare@nib.si; 6 Pennsylvania Cancer and Regenerative Medicine Research Center, Baruch S. Blumberg Institute, Pennsylvania Biotechnology Center, Wynnewood, PA 19096, USA; 7 Faculty of Chemistry and Chemical Technology, University of Ljubljana, 1000 Ljubljana, Slovenia

**Keywords:** *CCL5*, *CCR5*, chemokines, glioblastoma, invasion, maraviroc, mesenchymal stem cells

## Abstract

The chemokine *CCL5*/RANTES is a versatile inflammatory mediator, which interacts with the receptor *CCR5*, promoting cancer cell interactions within the tumor microenvironment. Glioblastoma is a highly invasive tumor, in which *CCL5* expression correlates with shorter patient survival. Using immunohistochemistry, we identified *CCL5* and *CCR5* in a series of glioblastoma samples and cells, including glioblastoma stem cells. *CCL5* and *CCR5* gene expression were significantly higher in a cohort of 38 glioblastoma samples, compared to low-grade glioma and non-cancerous tissues. The in vitro invasion of patients-derived primary glioblastoma cells and glioblastoma stem cells was dependent on *CCL5*-induced *CCR5* signaling and is strongly inhibited by the small molecule *CCR5* antagonist maraviroc. Invasion of these cells, which was enhanced when co-cultured with mesenchymal stem cells (MSCs), was inhibited by maraviroc, suggesting that MSCs release *CCR5* ligands. In support of this model, we detected *CCL5* and *CCR5* in MSC monocultures and glioblastoma-associated MSC in tissue sections. We also found *CCR5* expressing macrophages were in close proximity to glioblastoma cells. In conclusion, autocrine and paracrine cross-talk in glioblastoma and, in particular, glioblastoma stem cells with its stromal microenvironment, involves *CCR5* and *CCL5*, contributing to glioblastoma invasion, suggesting the *CCL5*/*CCR5* axis as a potential therapeutic target that can be targeted with repositioned drug maraviroc.

## 1. Introduction

Glioblastoma is one of the most aggressive brain tumors and poorly responsive malignancies to treatment with among the shortest survival rates of all cancers [1]. Patients’ 5-year survival rate is less than 5% [2], regardless of novel modalities in surgery, irradiation, and chemotherapy [3,4].

A high rate of relapse is mostly due to the resistance of glioblastoma stem cells (GSCs) and their heterogeneity and plasticity that are contributing to the resistance of recurrent tumors [3,5,6]. Tumor heterogeneity is due to both the variety of glioblastoma subtypes [7,8] and the presence of different types of stromal cells of the tumor microenvironment (TME), both contributing to the inter- and intra-tumor heterogeneity, respectively [9,10]. GSCs have an important role in the development, growth, and aggressiveness of glioblastoma, due to their efficient DNA repair mechanisms, heterogeneity, cell plasticity [5], and cooperation with differentiated glioblastoma cells [11]. In tumor tissues, GSCs are maintained in hypoxic and peri-arteriolar GSC niches [12,13] where the TME contributes to paracrine interactions with endothelial cells [12]. The brain TME is comprised not only of resident astrocytes, neurons, and microglial cells but also infiltrating mesenchymal stem cells (MSCs), hematopoietic stem cells (HSC), and differentiated immune cells, such as macrophages [9], altogether comprising so-called tumor stroma. Understanding these selective interactions between non-cancerous so-called stromal and glioblastoma cells is crucial for the effectiveness of treatment strategies.

These cellular interactions are mediated by proteins, called cytokines, of which a large family of chemokines is mediating chemoattraction between cells [14,15]. For example, the attraction between endothelial and glioblastoma cells is maintained by CXCL12 (SDF-1α) binding to the CXCR4 receptor [12] and cytokines, expressed by tumor or stromal cells, best known for their part in mediating leukocyte trafficking and lymphoid tissue development [16,17]. Outer cell membrane-bound chemokine receptors that all belong to the broader family of G-protein coupled receptors (GPCR), bind usually to a related group of chemokines that have a significant role in in the progression of cancer, in the shaping of the tumor microenvironment and is involved in invasion and metastasis [17]. Moreover, chemokines have an affinity for several chemokine receptors. The chemokine *CCL5*, originally termed RANTES (regulated on activation, normal T cell expressed, and secreted) is a CC chemokine ligand 5, both known for his role in inflammatory diseases and cancer progression [8,18,19,20]. The chemokine *CCL5* also binds to CCR1, CCR3, and *CCR5* chemokines receptors (C-C motif receptors 1, 3, and 5) and to the G protein-coupled receptor 75 (GPR75) [21,22]. *CCR5*/*CCL5* signaling has been extensively described by us [8,19,20,22] and others [18,21] and acts via calcium signaling. The role of both *CCL5* and *CCR5* has been elucidated in many types of cancers, expressed by cancer cells as well as non-cancerous cells in the TME [21,23,24,25]. In glioblastoma high levels of *CCR5*, CXCR4, CXCR7, CCR7, and CCR10 are linked to poor prognosis [26].

The first evidence that chemokines and receptors, like CCR3 and *CCR5* in human glioblastoma, may promote cell growth, was proposed by Kouno et al. [27] in 2004. In 2015, Zhao et al. [28] suggested a potential role of *CCR5* receptor in glioblastoma proliferation and invasion as *CCR5* was over-expressed during glioma progression to glioblastoma, correlating with reduced progression-free and overall survival [28]. Moogooei et al. [29] reported that *CCL5* (and CCL2) were elevated in serum and tissues of glioblastoma patients at both mRNA and protein levels, and proposed these chemokines as predictors for disease severity and response to treatment. However, the authors suggested that the main sources of circulatory and tissue *CCL5* were likely activated macrophages and T cells, which may contribute to the tumor expansion. The evidence that interactions between *CCL5* and *CCR5* guide infiltration of monocytes, macrophages, and MSCs into tumors, has recently been reviewed [8]. Thus *CCL5*-receptors signaling can favor cancer progression either directly by affecting proliferation, migration, and cell survival of cancer cells, or indirectly, by recruiting pro-tumor and/or anti-inflammatory effector cells. Yet the key relevance of autocrine vs. paracrine *CCL5*/*CCR5* singling axis in glioblastoma progression remains poorly understood and was therefore addressed in this study. 

Here, we hypothesized a correlation between *CCR5* and *CCL5* protein levels in individual patient-derived glioblastoma tissues, with respect to *CCR5* vs. *CCL5* distribution. We also explored the distribution of *CCR5* and *CCL5* among gliomas of different stages. Addressing the inter-tumoral heterogeneity of glioblastomas, using gene analyses, we defined four distinct glioblastoma subtypes [7]: the proneural (PN), mesenchymal (MES), neural (N), classical (CL), and mixed GB subtype, where two or more subtypes are present within a single tumor [10]. As these subtypes reportedly differ in survival rate, being the shortest in the MES subtype, and as cancer invasion was found associated with *CCL5*/*CCR5* axis signaling, we hypothesized that *CCR5* and/or *CCL5* distribution would be significantly different in GB subtypes. As the role of *CCL5*/*CCR5* expression in glioblastoma stem cell expansion had not been investigated, we investigated the role of *CCR5* expression in tumor invasiveness. Herein, the synthetic *CCR5* small molecule inhibitor maraviroc, currently in clinical trials targeting metastatic breast and colon cancer [22], was tested here for inhibition of glioblastoma invasion. Finally, we addressed glioblastoma intra-tumor heterogeneity, due to stromal cells’ interactions by analyzing *CCL5*/*CCR5* expression in tumor-associated macrophages and tumor-associated MSCs. We show that MSC enhances glioblastoma and stem cell matrix invasion via *CCR5*.

## 2. Results

### 2.1. Heterogeneous Expression of CCL5/CCR5 Axis in Glioblastoma Tissue Sections

**Figure 1:**
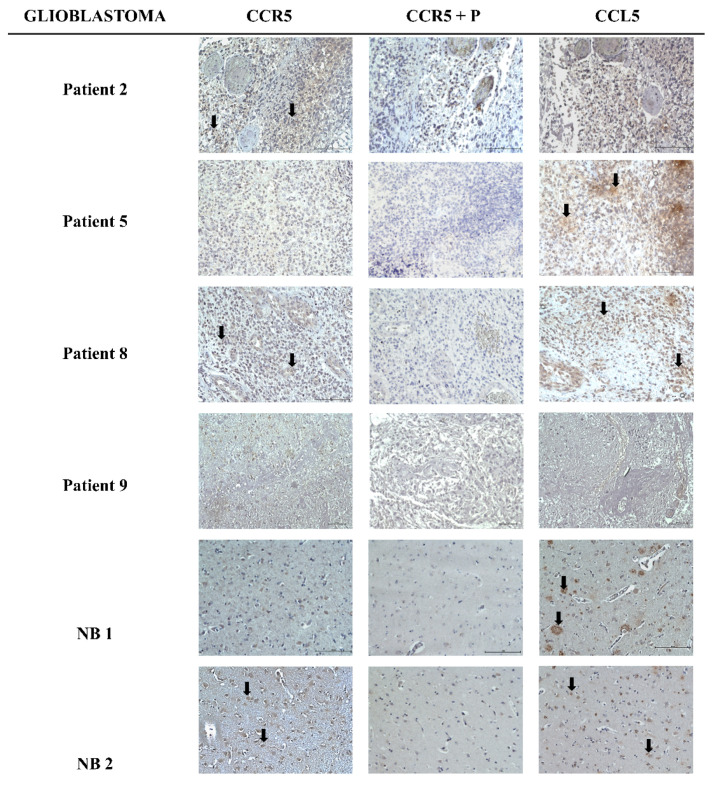
Brain tissue sections immunolabeling for *CCL5* and *CCR5*. Immunohistochemical localization of *CCL5* and *CCR5* in glioblastoma and non-cancerous tissue (NB1 and NB2) sections was performed as described in Materials and Methods. Cell nuclei were counterstained by hematoxylin (blue). *CCR5* epitope blocking peptide (P) was used (in *CCR5*+P images) as a control for specific binding of the primary antibody. Scale bar represents 100 µm. Black arrows indicate examples of *CCL5* and *CCR5* positive cells. Microscopy was carried out at 20× objective magnification.

Glioblastoma tissue sections were obtained from the Institute of Pathology, Medical Faculty, University of Ljubljana. To confirm the expression of *CCL5*/*CCR5* signaling axis, we performed immunohistochemistry (IHC) on a total of 8 tissue sections of GB patients, of which 4 are shown along with two non-cancer brain sections (NB1 and NB2) in Figure 1. In tissue sections, *CCR5* was expressed in 50% of the cases in around 30% of the cells. Non-cancer sample NB2 had a weak expression of *CCR5* in more than 33% of the cells, while the other NB1 sample had no expression of *CCR5*. *CCL5* was more abundant, expressed in 50% of brain tissue samples with strong intensity, including NB1 and NB2 samples. The sample patient Nb. 8 had a strong expression of *CCL5* and weak expression of *CCR5*. Sample patient Nb. 9 was negative for both *CCR5* and *CCL5*. Both proteins were localized in the cytoplasm of tumor-associated astrocytes. The quantification of IHC analyses is presented in Table 1. Clinical and histological parameters of glioblastoma patient samples, used in this study, are presented in Table 2.

### 2.2. Expression of CCL5/CCR5 Axis in Primary Glioblastoma Cells and Glioblastoma Stem Cells

**Figure 2:**
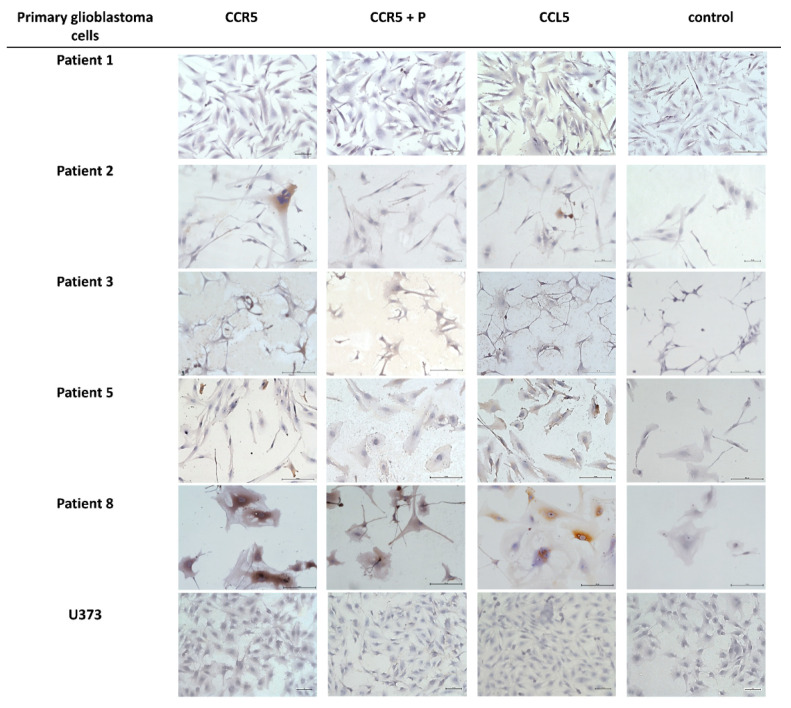
Immunocytochemical localization of *CCL5* and *CCR5* in primary glioblastoma cells. ICC localization of *CCL5* and *CCR5* in primary glioblastoma cells isolated from patients’ tumors and the glioblastoma cell line U373 as performed as described in Materials and Methods. Cell nuclei were counterstained by hematoxylin (blue). *CCR5* epitope blocking peptide (P) was used (in *CCR5* + P images) as a control. Negative control staining was performed in the absence of the primary antibody. Scale bar represents 50 µm. Microscopy was carried out at 20× objective magnification.

**Figure 3:**
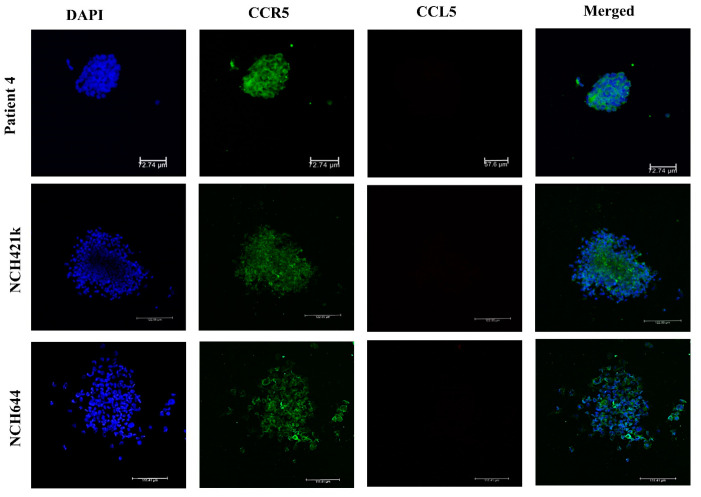
Primary glioblastoma stem cells express *CCR5*. Immunofluorescence labeling was performed as described in Materials and Methods on GSC spheroids, established from the patient’s Nb.4 tissue and established GSC lines NCH644 and NCH421k. Nuclei were stained with DAPI (blue), *CCR5* expression is shown as a green and *CCL5* as red fluorescence. The last panel presents merged channels. Confocal microscopy was carried out at 20× objective magnification. Scale bar represents 100 µm.

To further investigate the cellular origin of *CCL5* and *CCR5* in glioblastoma tissues, using IHC we screened for the expression of *CCL5* and *CCR5* in primary differentiated glioblastoma cells and glioblastoma stem cells (GSCs) that were cultured from patients’ tumors. Brain tissue samples from glioblastoma patients were obtained from the Department of Neurosurgery of the University Medical Centre, University of Ljubljana. These tumor samples were either used for the generation of primary glioblastoma cells and GSC or were frozen upon tumor removal for RNA extraction. GSC cells and the two previously established CD133+ GSC lines, NCH644, and NCH421k were grown as spheroid suspensions in serum-free, complete Neurobasal Medium as described by Podergajs [30]. Spheroids were fluorescently labeled for *CCL5* and *CCR5* expression as described in Materials and Methods. The ICC analyses are shown in Figure 2. The quantification scoring analyses revealed that the chemokine expression seems to be higher compared to the receptor and that very low or no *CCL5* nor *CCR5* expression was observed in patient Nb. 3 and in the U373 cell line (Table 3). In the three glioblastoma stem cells (GSCs) spheroids, high *CCR5* protein expression was seen, but *CCL5* could not be detected even using more sensitive detection by immunofluorescence (Figure 3). Normal astrocytes do not express *CCL5* nor *CCR5* (Figure S2).

### 2.3. Mesenchymal Stem Cells In Vitro and In Vivo Tumor Sections Express CCL5

**Figure 4:**
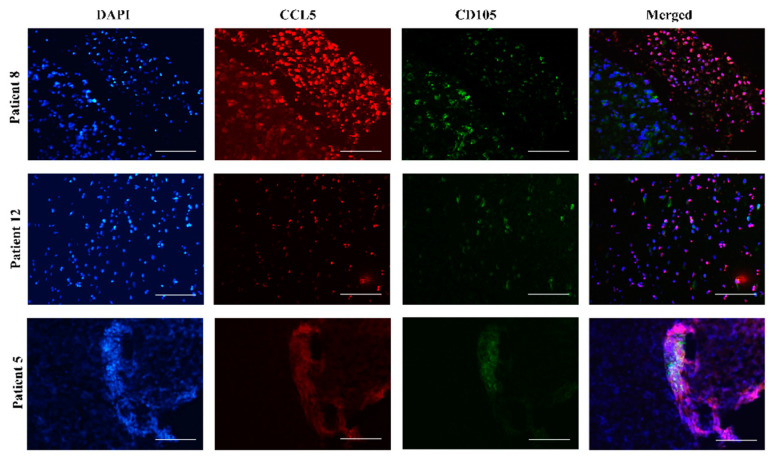
Mesenchymal stem cells in glioblastoma tissues express *CCL5*. Fluorescence immunohistochemical staining of *CCL5* antigen was performed on glioblastoma sections of 3 patients, Nb. 8, Nb. 12 and Nb. 5. MSCs were immunolabeled using the antibody against their specific marker CD105. Nuclei were stained with DAPI (blue), *CCL5* with Alexa Fluor 546 (red), and CD105 with Alexa Fluor 488 (green) dye. Merged images represent colocalization (violet color) of CD105 and *CCL5*. Microscopy was carried out at 20× objective magnification. Scale bar represents 100 µm.

The tumor progression of glioblastoma induces a host response, which is associated with the infiltration of stromal cells, e.g., bone-marrow-derived mesenchymal stem cells (MSC) and hematopoietic stem cells (HSC) and their progenitors, comprising various mature lymphocytes, macrophages [12,31]. Previous studies have shown that MSCs, homing to glioblastoma can de-differentiate to other stromal cells via paracrine effectors, such as immunomodulatory cytokines, or by direct interactions with GB cells [32]. Moreover, we have demonstrated that human MSCs exploit the immune response mediating chemokines to impact the phenotype of glioblastoma [32,33] and later described complex mechanisms of their indirect [14,34] and/or direct cross-talk [35,36]. Here, we were interested if MSC were alone and when in glioblastoma microenvironment express *CCL5* and *CCR5*. Therefore, we have immunolabeled bone marrow-derived MSCs in monocultures by *CCL5* and *CCR5* antibodies and demonstrated high expression of both antigens in MSCs (Figure S1). Furthermore, labeling these antigens of tissue sections from 3 patients, (Nb. 8, Nb. 12, and Nb. 5), we found that *CCL5* expression was co-localized with MSC marker CD105 (Figure 4).

### 2.4. CCR5 Is Expressed in Glioblastoma-Associated Macrophages in Tumors

**Figure 5:**
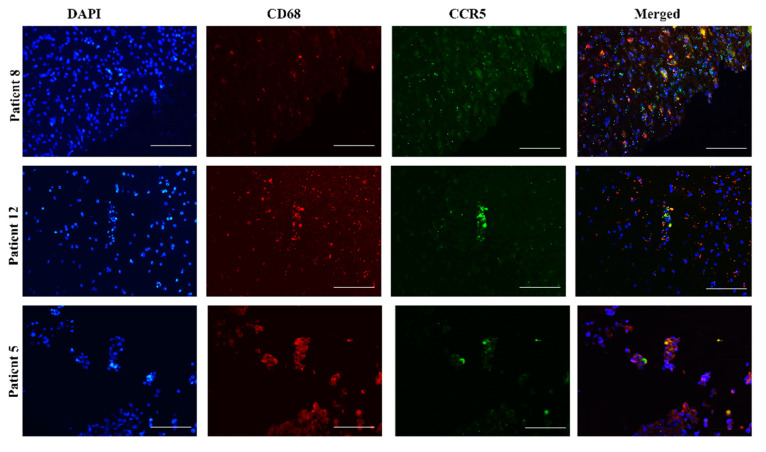
Glioblastoma-associated macrophages express *CCR5*. Fluorescence immunohistochemical staining of *CCR5* antigen was performed on glioblastoma sections of 3 patients, Nb. 8, Nb. 12 and Nb. 5. Macrophages were immunolabeled, using an antibody against the specific marker CD68. Nuclei were stained with DAPI (blue), CD68 with Alexa Fluor 546 (red), and *CCR5* with Alexa Fluor 488 (green) dye. Merged images represent colocalization (yellow color) of CD68 and *CCR5*. Microscopy was carried out at 20× objective magnification. Scale bar represents 100 µm.

Cross-talk of glioblastoma cells with microglia and infiltrating macrophages occurs through the release of cytokines, which promote tumor growth [14,35,37]. Tumor-associated macrophages represent about 40% of all cells in a glioblastoma specimen [38] and microglia-mediated immunosuppression may involve *CCL5*/*CCR5* via *CCR5* signaling on macrophages to induce their activation and polarization [39]. Fluorescence immunohistochemical staining of tissue sections of 3 patients (Nb. 8, Nb. 12, and Nb. 5) revealed the expression of *CCR5* in glioblastoma-associated macrophages, labeled by the antibody specific marker CD68 (Figure 5). 

### 2.5. CCL5 and Mesenchymal Stem Cells Enhance the Invasion of Primary Glioblastoma Cells and Glioblastoma Stem Cells

Glioblastoma cell invasion that has been characterized as a single cell infiltration into the brain parenchyma is crucially supported by its microenvironment comprised of stromal cells. Previously, we have mostly studied mesenchymal stem cells (MSCs), affecting glioblastoma cell phenotype via paracrine interactions, secreting cytokines as demonstrated by Motaln et al. [34] and Breznik et al. [36]. Here, we focused on revealing the functional significance of the *CCL5*/*CCR5* axis in paracrine, i.e., indirect MSC-GB cell interaction, such as invasion. Transwell chambers invasion assays were used to investigate primary GB cells from patient Nb. 2, which expressed high *CCR5*, but very low *CCL5* antigens (Figure 2). 

**Figure 6:**
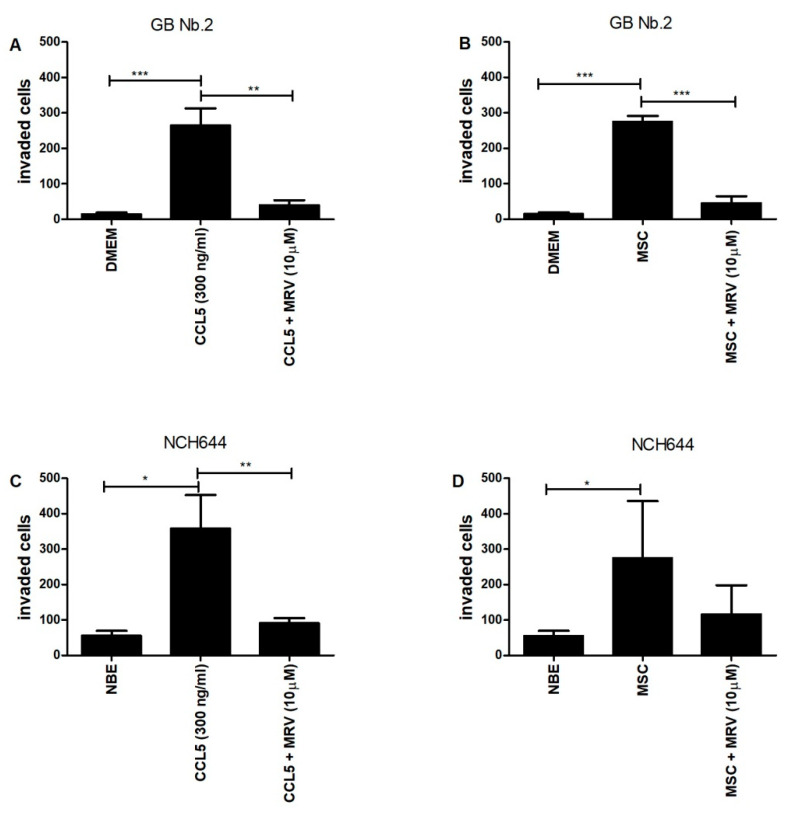
Effects of *CCL5* and MSC on the invasion of primary glioblastoma cells and glioblastoma stem cells. Primary glioblastoma cells from patient Nb. 2 (GB Nb.2) (10,000 cells/insert) and GSC cells (NCH644) (80,000 cells/insert), were seeded in the upper compartment alone or in combination with maraviroc (MRV) (final concentration 10 µM) which was coated with 0.5 mg/mL Matrigel in serum-free medium. (**A**,**C**) Recombinant *CCL5* (final concentration 300 ng/mL) was added to the lower chamber (**B**,**D**) MSCs (20,000/insert) were added to the lower chamber. The cells that invaded the matrigel after 48 h, were stained with 0.1% crystal violet and counted using an inverted microscope. Each value represents mean ± SD (*n* = 3). ^★^ *p* < 0.05, ^★★^ *p* < 0.01, ^★★★^ *p* < 0.001 vs. control group (*t*-test).

After the cells were stimulated with recombinant human chemokine *CCL5*, added to the lower chamber, Matrigel invading cells were quantified as described in Methods. The invasion was inhibited by synthetic *CCR5* antagonist maraviroc added to the cells in the upper chamber (Figure 6A). When *CCL5*-expressing MSCs (Figure S1) were added to the lower transwell compartment as a chemoattractant, GB Nb.2 cells’ invasion was significantly enhanced and was also inhibited by maraviroc (Figure 6B). Noteworthy, GB Nb.2 cell viability was not affected by maraviroc even at higher concentrations (Figure S3). Further, we validated the functional role of the *CCL5*/*CCR5* axis in the invasion of established GSC line NCH644, which expresses *CCR5* but low or no *CCL5* (Figure 3). GSCs were stimulated with recombinant human chemokine *CCL5* and their invasion in the presence or absence of maraviroc was quantified as above (Figure 6C). When *CCL5*-expressing MSCs were used as a chemoattractant in the lower chamber, GSCs invasion was significantly enhanced but was remarkably inhibited by the *CCR5* inhibitor maraviroc (Figure 6D). The significance was not reached here, due to the higher variance among biological repetitive experiments.

### 2.6. CCL5 and CCR5 mRNA Levels Are Increased in High-Grade Gliomas

To determine, if the high protein levels of *CCL5*/*CCR5* in GB tissues result from increased gene expression, i.e., transcriptional activity, we determined the mRNA levels of *CCL5* and *CCR5* in the tissues of normal and malignant specimens: non-cancerous brain tissues (*n* = 16), glioma I-II- low-grade gliomas (*n* = 17), glioma III-anaplastic astrocytomas (*n* = 5), glioma IV-GBs (*n* = 38), recurrent GB (GB rec) (*n* = 5), GB cells-primary glioblastoma cells (*n* = 10) and GSCs (*n* = 6), isolated from patient tumor samples). 

**Figure 7:**
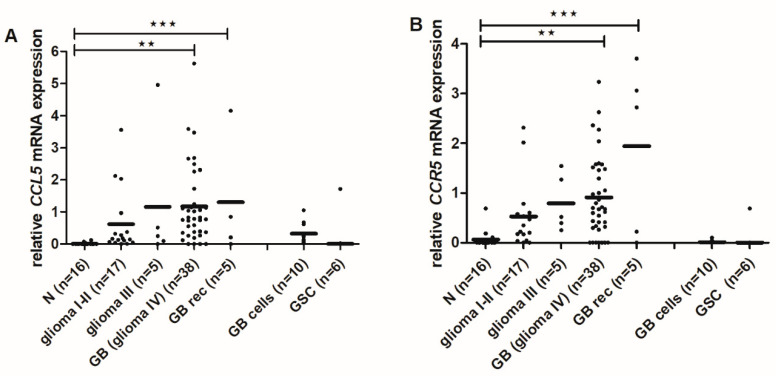
*CCL5* and *CCR5* gene expression in glioma tissues and primary glioblastoma cells. The expression of *CCL5* (**A**) and *CCR5* (**B**) at mRNA was determined in glioma, non-cancerous brain tissues and glioblastoma cells analyzed by RT-qPCR. mRNA values were normalized to housekeeping genes *HPRT1* and *GAPDH* and analyzed with quantGenius software [40] as described in Materials and Methods. n-number of samples; N-non-cancerous brain tissues; glioma I-II- low-grade gliomas: pilocytic astrocytoma, astrocytoma, oligodendroglioma; glioma III-anaplastic astrocytoma, anaplastic oligodendroglioma, and anaplastic mixed oligoastrocytoma; GB-glioblastoma; GB rec- recurrent glioblastoma; GB cells-primary glioblastoma cells; GSC-glioblastoma stem cells isolated from patient tumor samples. ^★★^ *p* < 0.01, ^★★★^ *p* < 0.001 versus the non-cancerous brain tissues.

*CCL5* and *CCR5* mRNA levels were significantly higher in GB and GB rec samples compare to non-cancerous brain tissues (Figure 7A,B). Primary glioblastoma cells expressed higher levels of *CCL5* mRNA, compared to GSCs what also correlated with protein levels seen in Figure 2 and Figure 3.

### 2.7. CCL5 and CCR5 mRNA Levels Differ among Glioblastoma Subtypes

**Figure 8:**
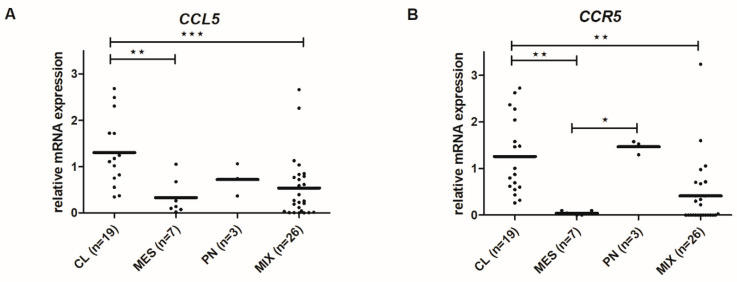
*CCL5* and *CCR5* gene expression in glioblastoma subtypes. mRNA expression of *CCL5* (**A**) and *CCR5* (**B**) in glioblastoma tissues and glioblastoma cells analyzed by RT-qPCR. mRNA values were normalized to housekeeping genes *HPRT1* and *GAPDH* and analyzed with quantGenius software as described in Materials and Methods. n-number of samples; CL-classical, MES-mesenchymal, PN-proneural, and MIX subtypes. ^★^ *p* < 0.05, ^★★^ *p* < 0.01, ^★★★^ *p* < 0.001 versus the non-cancerous brain tissues.

We analyzed the *CCL5* and *CCR5* mRNA levels in four GB subtypes, MES (Mesenchymal), PN (Proneural), CL (Classical), and MIX, based on the expression values of 12 genes from Behnan et al. (2016) [41]. PN subtype was classified with expression levels of *P2RX7, STMN4, SOX10,* and *ERBB3* genes. CL subtype was classified with expression levels of *ACSBG1* and *KCNF1* and MES subtype with expression levels of *S100A, DAB2, TGFB1, THBS1, COL1A2,* and *COL1A1*, as described in Materials and Methods. Cl subtype exhibited the highest level of both *CCL5* and *CCR5* mRNA expressions, while MES expressed the lowest (Figure 8).

## 3. Discussion

The *CCL5*/*CCR5* axis has been reported as a mechanism of tumor progression in pancreatic [19], gastric [23], and breast cancer [42]. The *CCL5*-receptors’ signaling can favor cancer progression, directly affecting proliferation, migration, and cell survival of cancer cells by autocrine signaling, or indirectly by paracrine signaling recruiting pro-tumor and/or anti-inflammatory effector cells into the tumor microenvironment (TME) [43].

The basic question when investigating chemokine autocrine signaling in cancer, such as presented by *CCL5*/*CCR5* axis, is “what activates what,” whereas in paracrine signaling in heterogeneous cancers the question is “what attracts what.” Autocrine signaling means that GB cells express both ligand and receptor, and thus activate the pathways downstream of *CCR5* in a cell-autonomous manner. Therefore, we first need to reveal the *CCL5*/*CCR5* distribution in patients’ tissues with respect to glioma stage and glioblastoma subtype, and secondly *CCL5* and *CCR5* relative expressions in the isolated primary glioblastoma cell lines. By analyzing *CCL5*/*CCR5* mRNA and protein expression in glioma tissues in a larger cohort of 65 patients, we confirmed that both, *CCL5* and *CCR5* genes are increasingly expressed in advanced glioma (Figure 7). Moreover, higher *CCL5* and *CCR5* were detected in secondary, recurrent glioblastoma as compared to the primary glioblastoma. As the recurrences are known to be more aggressive [44], we suggest that *CCL5* and *CCR5* autocrine signaling is playing a significant role in glioblastoma progression. Secondly, we detected higher *CCL5* and *CCR5* proteins in glioblastoma tissues and cells than in non-malignant brain tissues and normal astrocytes (Figure 1 and Figure 2).

Our results are consistent with studies that showed increased *CCL5* and *CCR5* expression in human glioma tissues [45,46] and glioblastoma cells [47], compared to normal counterparts. Our finding of increased *CCR5* expression in worse prognosis glioma are consistent with reports that high *CCR5* levels correlate with shorter survival [28]. Similarly, *CCL5* was demonstrated as a bad prognostic marker for survival of patients with various types of cancer [19]. As in low-grade gliomas, for which we have shown low expression of both *CCL5* and *CCR5*, we may speculate that the progression probably relays more on stromal cells’ supportive chemokine stimulation. Whereas in glioblastoma, high levels of *CCL5*/*CCR5* enable an autocrine chemokine activation, resulting in increased tumor cell proliferation and invasion [45,46] that is becoming independent of stromal cells.

This is leading to a lower survival rate of glioblastoma patients, as suggested by Pan et al. [45]. These authors showed that *CCL5* established autocrine signaling in high-grade glioma by affecting growth regulatory circuit that was critical in particular for mesenchymal (MES) glioblastoma subtype. In contrast to expected, in our group of patient-derived GB tissues, *CCL5* and *CCR5* proteins were not correlating, as each may be present in some specimen, but absent in others, and they were found in a different subcellular compartment. This indicates on non-exclusive partnering, but also the promiscuous binding of *CCL5* and CRR5 in tumors in vivo [45,47,48]. 

As mentioned above, recurrent glioblastoma supposedly acquires aggressive mesenchymal phenotype, being possibly induced by irradiation [44] via epithelial to mesenchymal transition (EMT). Such phenotype is more invasive and tends to express higher stemness-related genes (Majc et al., accepted, 2020) [49]. The dilemma of autocrine signaling in glioblastoma is therefore also related to glioblastoma cell subtypes. However, exploring the gene expression distribution of *CCR5* and *CCL5* among different genetic subtypes, we found the highest levels of both genes in the CL-glioblastoma subtype and the lowest in MES-glioblastoma subtype (Figure 8). This contrasts to the results by Pan et al. [45], demonstrating the highest *CCL5* (gene) expression in MES-glioblastoma and the lowest in PN-glioblastoma. This discrepancy could be due to the low number of samples in each subtype group in our study, and using smaller [41] vs. larger panel of gene fingerprints defining the subtype [7] by us than by Pan et al. [45]. Moreover, in MES-glioblastoma, the autocrine *CCL5*-dependent activation loop has also been proven by adding exogenous *CCL5*, and because no further activation was achieved, it was concluded that *CCL5* promotes survival and proliferation of the cells in a cell-autonomous manner. Noteworthy, MES-subtypes characteristically express the CD44 a non-conventional *CCL5* receptor, also a stemness marker [50]. As *CCL5* is a promiscuous ligand, binding to more than one receptor [46], several receptors need to be blocked to inhibit *CCL5* driven axis processes in brain tumors. 

There are three major reasons for poor survival: (1) increased tumor cell invasion, (2) the abundance of more aggressive glioblastoma stem cells (GSCs), and (3) supportive stromal cells in TME. Firstly, glioblastoma invasion is characterized by extensive single-cell infiltration into healthy brain tissue, preventing total tumor removal during surgery [51]. Increased invasion of glioblastoma cells could also be activated by *CCL5*/*CCR5* signaling the migratory downstream pathways through αvβ3 integrin, PI3K/Akt kinases, NF-κB pathways [52], and proteases such as matrix metalloproteases (MMPs) [53]. However, protease inhibitors, such as MMPs and cathepsin inhibitors failed to inhibit invasive cancer spread in clinical trials. Thus *CCL5*/*CCR5* axis blocking agents were suggested as efficient anti-invasive therapeutics [54]. Both ligand *CCL5* and its receptor *CCR5* have been suggested as potential therapeutic targets in various cancers, including glioblastoma, breast and prostate cancer, and impairing disease progression [28,55]. Most promising is *CCR5* blocking by synthetic drug maraviroc, an allosteric inverse *CCR5* agonist [56], which has been proven to significantly inhibit proliferation, colony formation, and migration of several carcinomas, including breast [42] and prostate cancer [55]. Maraviroc has very recently been reported also in metastasis of breast cancer cells xenografts [18,20,22,42]. Here, we demonstrated that *CCR5*-expressing primary glioblastoma cells and glioblastoma stem cells (GSC) invasion, when enhanced by recombinant *CCL5*, was also significantly inhibited by adding maraviroc. 

Secondly, we demonstrated that maraviroc inhibited glioblastoma stem cell (GSCs) invasion. This is an important novelty of this research, as GSCs are recognized as a key target of therapy in glioblastomas and all other cancers, as these are cancer stem cells (CSCs) and are highly resistant to irradiation and chemotherapy. As CSCs represent the tumor-initiating cells, i.e., the seed of primary and the secondary tumors metastases, these are the cells that need to be eradicated by a novel kind of therapy. High levels of GSCs in glioblastoma were observed in more aggressive tumors vs. low-grade glioma, as reported by us and others [57,58] and their abundance is related to prognosis.

These cells are trafficking within the tissues into and out their niches [12] and presumably invade into the brain parenchyma, based on the chemoattraction among the tumor and stromal cells, as has been demonstrated for CXCR12/SDF-1α [31,59]. As maraviroc significantly inhibited *CCL5*-induced GSC invasion (Figure 6), we propose targeting *CCL5*/*CCR5* signaling as novel glioblastoma therapeutics, as initially suggested by Kast et al. [60]. Moreover, we show that GSCs express only *CCR5*, but not *CCL5* (Figure 3), indicating that only paracrine signaling would stimulate GSC invasion. This may be occurring in vivo, as dormant GSCs reside in glioblastoma tissue niches and are presumably activated in a paracrine cross-talk by stromal cells, infiltrating the niche to migrate out of the niches [12,59], expressing *CCL5* and *CCR5*. 

Thirdly, glioblastoma TME consists beside brain tissue astrocytes and microglia, also from infiltrating immune cells, macrophages, lymphocytes, neutrophils, and mesenchymal stem cells (MSCs) that interact in complex networks of molecular signals [43,61], where chemokines are the key molecules for directing the cells to move along a chemical gradient towards the tumor [62]. We are still far from understanding complex multiple interactions under in vivo conditions, however, by categorically studying bilateral ligand and receptor expressions by selected cell types, their specific mechanisms in *CCL5*/*CCR5* signaling in glioblastoma may be elucidated. Here, we focused on MSCs, proven as glioblastoma-infiltrating cells, recruited from bone marrow or brain tissues, and also present in GSC niches, where MSCs may also affect glioblastoma cell differentiation and proliferation as well as invasion, as proven by us and others [12,31,36,63].

We demonstrated that paracrine MSC-glioblastoma and GSC cell interactions enhance invasion, maintained by *CCR5* receptors, as it was inhibited when maraviroc was added to the system (Figure 6). Our extensive previous research [35,36,64], provided sufficient evidence by quantifying a set of chemokines released from bone-marrow MSCs in indirect co-cultures and glioblastoma cells. MSC have been demonstrated to secrete among other chemokines, also *CCL5*, which interacts with specific cytokine receptors such as CCR1, CCR3, and *CCR5*. *CCL5* paracrine signaling was found to promote the migratory, invasive, and metastatic properties of breast cancer cells [24]. Similar was later confirmed by Choi et al. [65] demonstrating that also adipose MSCs target brain tumor-initiating cells from glioblastoma, medulloblastoma, and ependymoma, by releasing potential cytokines, including CXCR4/SDF-1alpha, *CCR5*/RANTES, IGF1R/IGF-1, IL6R/IL-6, and IL8R/IL-8.

Complementary to this, we demonstrated here the bilateral ligand and receptor expression on glioblastoma tissue sections using the specific markers of CD105 for MSCs and CD68 for macrophages. We showed that MSCs express ligand *CCL5* (Figure 4) and macrophages receptor *CCR5* (Figure 5). These results further suggest that the *CCL5*/*CCR5* axis may mediate cellular cross-talk between MSCs, macrophages, and GSCs by attracting them to peri-vascular tumor niches, that are populated by MSCs. The involvement of the *CCL5*/*CCR5* axis in MSC-GB cell interactions has not been known so far, in comparison to the well-known pro-migratory role of macrophage-secreted *CCL5* [53]. Finally, we hypothesize that MSC-secreted *CCL5* maintains the interactions between MSCs and GSCs, and targeting the *CCL5*/*CCR5* axis with maraviroc may become effective anti-invasive therapy preventing invasive GSCs migration out of their niches to spread to brain parenchyma. 

In conclusion, we have demonstrated the heterogeneous tissue/cellular distribution and subcellular expression of *CCL5* and *CCR5* in glioblastoma. Using the *CCR5* antagonist, maraviroc, we have shown *CCL5* and *CCR5* drive primary glioblastoma (GB) cells and glioblastoma stem cells (GSCs) invasion and their interactions with stromal MSCs and can be used as repositioned drug for novel clinical trials in glioblastoma. These results suggest paracrine and autocrine *CCL5*/*CCR5* axis-dependent signaling in a lower grade (gliomas) vs. higher grade glioblastoma invasion. The potential role of *CCL5*/*CCR5* in paracrine GSC niche interactions warrants further investigations. 

## 4. Materials and Methods 

### 4.1. Cell Cultures

Human bone marrow-derived MSCs were obtained from Lonza Bioscience (Walkersville, MD, USA; Lot139 number 6F4393). MSCs were cultured in Dulbecco’s medium (DMEM 5921; Sigma-Aldrich, St. Louis, MO, USA) containing 10% (*v/v*) heat-inactivated Fetal Bovine Serum (FBS, Gibco, Dublin, Ireland), 100 IU/mL penicillin (Thermo Fisher Scientific, Waltham, MA, USA), 100 µg streptomycin (Thermo Fisher Scientific, Waltham, MA, USA), 2 mM L-glutamine (Thermo Fisher Scientific, Waltham, MA, USA), sodium- pyruvate (Gibco, Dublin, Ireland), and nonessential amino acids (Sigma-Aldrich, St. Louis, MO, USA ). Human glioblastoma cell line U373 cells were obtained from American Type Culture Collection (ATTC, Manassas, VA, USA) and were grown in DMEM high glucose medium (GE Healthcare, Il, Chicago, IL, USA), supplemented with 10% (*v*/*v*) FBS, 2 mM L Glutamine, 100 IU/mL penicillin and 100 µg streptomycin, as described in Kološa et al. [63] and Breznik et al. [36]. Glioblastoma stem cell lines, NCH644 and NCH421k were obtained from CLS (Cell Lines Service GmbH, Eppelheim, Germany) and grown as spheroid suspensions in complete Neurobasal Medium (Invitrogen, Life Technologies, Carlsbad, CA, USA) containing 2 mM L-glutamine, 1 × penicillin/streptomycin, 1 × B-27 (Invitrogen, Life Technologies, Carlsbad, CA, USA), 1 U/mL heparin (Sigma-Aldrich, St. Louis, MO, USA), 20 ng/mL bFGF and EGF (both from Invitrogen, Life Technologies, Carlsbad, CA, USA). All cell lines were maintained at 37 °C with 5% CO_2_ and 95% of humidity. All cell cultures were tested for mycoplasma contamination using MycoAlert Mycoplasma Detection Kit (Lonza, Basel, Switzerland).

### 4.2. Glioblastoma Samples from Patients

Glioma biopsies were obtained from 65 patients that operated at the Department of Neurosurgery, University Medical Centre of Ljubljana, Slovenia. Tumor tissue samples were snap-frozen in liquid nitrogen and stored in the liquid nitrogen for RNA/DNA analyses. The study was approved by the National Medical Ethics Committee of the Republic of Slovenia (approval no. 0120-179 190/2018/4). Patients with glioblastoma (glioma grade IV) were selected for this study (Table 2). The clinical parameters and tumor characteristics were provided by the Department of Neurosurgery and Institute of Pathology at medical faculty in Ljubljana (Table 2). Formalin-fixed, paraffin-embedded tissues were prepared at the Institute of Pathology and were used for immunohistochemical analyses. Non-cancer brain samples (NB1 and NB2) were also obtained from the Institute of Pathology, from patients who were brain cancer-free. 

### 4.3. Establishment of Primary Glioblastoma and Glioblastoma Stem Cell Lines 

Fresh glioblastoma tumor tissue samples were minced by scalpels in DMEM/high glucose cell culture media supplemented with 10% FBS, 2 mM L-glutamine, and penicillin-streptomycin and seeded in 6 well plates. Outgrowing cells were detached with 0.25% trypsin-EDTA solution (Sigma-Aldrich, St. Louis, MO, USA) and transferred to T25 cell culture flasks. Cells were collected by low-speed centrifugation (1000 rpm for 60 s). After centrifugation 2–3 times, the cells were transferred to T75 culture flasks and expanded for subsequent analyses.

Cells’ solution was further filtered through Nylon mash 40 μm pores (BD Falcon cell strainer, Nylon). Single cells were collected and resuspended in stem cell media, Neurobasal Medium (Invitrogen, Life Technologies, Carlsbad, CA, USA) containing 2 mM L-glutamine, 1 × penicillin/streptomycin, 1 × B-27 (Invitrogen, Life Technologies), 1 U/mL heparin (Sigma-Aldrich, St. Louis, MO, USA), 20 ng/mL bFGF and EGF (both from Invitrogen, Life Technologies, Carlsbad, CA, USA) and cultured on agar coated T25 flasks until spheres with a diameter of 200 μm were formed. Healthy spheres were frozen in stem cell media with 10% DMSO for further analysis. GSCs were authenticated for stem cell marker CD133 and SOX2 expression using immunofluorescence.

### 4.4. Immunohistochemistry and Immunocytochemistry

Immunohistochemistry (IHC) analyses were performed using antibodies against *CCR5* (ab65850, Abcam, Cambridge, UK), *CCR5* peptide (ab192862, Abcam, Cambridge, UK), and *CCL5*-RANTES (ab189841, Abcam, Cambridge, UK). After fixation, tumor sections (4 μm thick) were deparaffinized in xylene and rehydrated in ethanol. Antigen retrieval was carried out in 10 mM sodium citrate buffer (pH 6.0) at 95 °C for 20 min followed by 20-min cooling on ice. The sections were treated with 100% methanol (Merck, Kenilworth, NJ, USA) containing 0.3% H_2_O_2_ (Merck, Kenilworth, NJ, USA) for 10 min to block endogenous peroxidase activity to reduce non-specific background staining, followed by a washing step in distilled water. Non-specific binding sites were blocked with 1% bovine serum albumin with 2% goat serum in PBS before incubation with antibodies overnight in the fridge. The sections were incubated with biotinylated secondary antibody followed by horseradish peroxidase-conjugated streptavidin (Cell Signaling Technology, Danvers, MA, USA). The sections were further incubated with the 2,4-diaminobenzidine substrate and counterstained with hematoxylin. Immunocytochemistry was performed as described without the deparaffinization and antigen retrieval. To achieve high antibody specificity, we used *CCR5* blocking peptide (*CCR5* P) that binds specifically to the target antibody epitope in 10 times higher concentration as the primary *CCR5* antibody. IHC scoring was performed by a pathologist using a semi-quantitative grading system; immunostaining intensity: +++ strong, ++ moderate, + weak or no expression, and the abundance of stained cells percentage positive cells: 0, 1–33% = 1, 33–66% = 2, 66–100% = 3. The intracellular localization was evaluated as m = membrane, n = nuclear, c = cytoplasmic; e = extracellular.

### 4.5. Immunofluorescence of Glioblastoma Stem Cell Spheroids

The 3D GSC spheroids were washed with PBS, fixed in ice-cold methanol (Sigma-Aldrich, St. Louis, MO, USA) for 15 min at room temperature, and incubated for 15 min in 0.1% Triton X-100/1% FBS/PBS at room temperature for membrane permeabilization. The spheroids were stained for 30 min at room temperature with the following antibodies: *CCR5* (ab65850, Abcam, Cambridge, UK ) and *CCL5*-RANTES (ab189841, Abcam, Cambridge, UK). Negative control staining was performed in the absence of the primary antibodies. Spheroids were stained with an Alexa Fluor 488^®^- and Alexa Fluor 546^®^- conjugated secondary antibody (1:200; Invitrogen, Life Technologies, Carlsbad, CA, USA ) for 30 min at room temperature. For nuclear staining, the spheroids were incubated with the Hoechst 33342 dye (1:1000, Invitrogen, Life Technologies), for 5 min at room temperature. The spheroids were then mounted in AntiFade reagent (Invitrogen, Life Technologies, Carlsbad, CA, USA) and analyzed with a confocal microscope (Leica DFC 7000 T, Wetzlar, Germany). 

### 4.6. Immunofluorescence of Glioblastoma Tumor Tissue Sections

Tumor sections, prepared at the Institute of Pathology, Medical Faculty, were deparaffinized in xylene and rehydrated in ethanol. Following rehydration, antigen retrieval was carried out in 10 mM sodium citrate buffer (pH 6.0) at 95 °C for 20 min followed by 20-min cooling on ice. 

Non-specific binding sites were blocked with normal goat serum (Dako) and 0.1% Triton-X for 1 h at room T to reduce non-specific background staining. Sections were incubated overnight at 4 °C with primary antibodies, diluted in PBS containing 1% BSA (Sigma-Aldrich, St. Louis, MO, USA); *CCR5* (ab65850, Abcam, Cambridge, UK), *CCL5*-RANTES (ab189841, Abcam, Cambridge, UK), CD68 (Dako, clone EBM11), CD105 (ab27422, Abcam, Cambridge, UK), CD133 (ab19898, Abcam, Cambridge, UK) and GFAP (ab10062, Abcam, Cambridge, UK). The sections were washed in PBS containing 1% BSA. Alexa Fluor 488-conjugated goat anti-rabbit antibodies (Life Technologies, Carlsbad, CA, USA) and Alexa Fluor 546-conjugated goat anti-mouse antibodies (Thermo Fisher Scientific, Waltham, MA, USA), were used as secondary antibodies in 1:200 PBS dilution, containing 1% BSA for 1 h at roomT. Sections were washed in PBS for 5 min and coverslipped using Prolong Gold mounting medium with DAPI (Life Technologies, Carlsbad, CA, USA). Control incubations were performed in the absence of primary antibodies. Fluorescence imaging was performed using NIS-Elements AR 4.13.04 software and a Nikon Eclipse Ti-inverted microscope (Nikon Instruments, Melville, NY, USA). 

### 4.7. Invasion Assay

Primary glioblastoma cell (GB) and glioblastoma stem cell (GSC) invasion was measured using 24-well Transwell units with 6.5 mm inserts and 8 µm pores (Corning, New York, NY, USA). Primary GB from patient 2 (10,000/insert) and GSC (NCH644, 80,000/insert), were seeded in the upper compartment, which was coated with 0.5 mg/mL Matrigel (Becton Dickinson, Franklin Lakes, NJ, USA) in serum-free medium. The lower compartment was seeded with MSC (20,000/insert) in MSC media containing 10% FBS or with recombinant *CCL5*/RANTES peptide (R&D, 278-RN-050, Minneapolis, MN, USA) (300 ng/mL). Maraviroc (MVR, Selleckchem, S200, Houston, TX, USA) in a final concentration of 10 µM was added into the upper chamber to GB and to GSCs. Cells were allowed to invade at 37 °C in 5% CO2 for 48 h. Non-invading cells were removed from the upper surface of the membrane using a cotton swab. The lower surface of the membrane was fixed in 4% PFA, stained with 0.1% crystal violet, and stained cells were counted using the Nikon Eclipse Ti- inverted microscope (Nikon Instruments, Melville, NY, USA) at 4× magnification. Three biological experiments with two separate membranes for each condition were analyzed.

### 4.8. Cell Viability Assay

Cell viability of primary glioblastoma cells was determined after 48 h of treatment with maraviroc (MVR, Selleckchem, S2003, USA) using the MTT reagent (3-(4,5-dimethylthiazol- 2-yl)-2,5-diphenyltetrazolium-bromide; Sigma-Aldrich, USA) according to manufacturer’s instructions. Briefly, cells were seeded into 96-well plates (8000 cells/well) and grown overnight. Cells were treated with different concentrations of MVR (0.1–50 µM). Stock solutions of MVR were prepared in dimethyl sulfoxide (DMSO, Sigma-Aldrich, St. Louis, MO, USA). Control incubations contained the same amount of DMSO (0.2%, *v*/*v*). After 48 h, MTT was added and after 3 h of incubation, the formed formazan crystals were dissolved in DMSO and the absorbance was measured as the change in optical density (ΔOD 570/690 nm) using microplate reader (Synergy™ HT, Bio-Tec Instruments Inc., Winooski, VT, USA). Cell viability was analyzed using GraphPad Prism software (GraphPad Software, San Diego, CA, USA)

### 4.9. Gene Expression Analysis

Total RNA from glioblastoma tissues and cells was isolated using AllPrep DNA/RNA/Protein Mini Kit (Qiagen, MD, USA) according to the manufacturer’s instruction. 1 µg of RNA was reverse transcribed using a High-Capacity cDNA Reverse Transcription Kit (Thermo Fischer Scientific, Waltham, MA, USA). High-throughput RT-qPCR was used to measure *CCL5*, *CCR5* expression. RT-qPCR was performed with FAM-MGB probes with Fluidigm BioMark HD System RT-PCR (Fluidigm Corporation, San Francisco, CA, USA) using 48.48 Dynamic Arrays IFC [66], where 42 samples and 24 assays (probes) were mixed pairwise in nanoliter chambers to enable parallel analysis of 2304 reactions. 

Visualization and analysis of qPCR results were done using the Fluidigm RT-qPCR analysis software and quantGenius software [40]. Relative copy numbers of mRNA were normalized to housekeeping genes HPRT1 and GAPDH. Assays are described in Table S1.

### 4.10. Data Analysis

#### 4.10.1. Glioblastoma Subtyping

Firstly, we assessed whether the expression profiles of 12 selected genes (*COL1A2, COL1A, TGFB1, THBS1, DAB2, S100A4, P2RX7, STMN4, SOX10, ERBB3, ACSBG1, KCBF1*) from 4 sample types (GB-glioblastoma; GB rec-recurrent glioblastoma; GB cells-primary glioblastoma cells; GSC-glioblastoma stem cells) are suitable markers for GB subtype distinction into mesenchymal (MES), proneural (PN), classical (CL) subtype and finally the subtype combination (MIX). Since the number of subtypes (clusters) was known in advance, we used k-means clustering to partition the expression profiles of the selected genes in one of the four subtypes. K-means clustering partitions each gene to the subtype (cluster) with the nearest mean. Data was first standardized. We used two clustering techniques, k-means, and PAM (partition around medoids). 

The difference between the two is that k-means uses artificially calculated means, while PAM uses the so-called medoids, which are actual dataset values. PAM is also more robust. The cluster (subtype) assignment for each gene was then compared and the method which shows more concordance with the subtype assignment from clinical data (EGFRIII mut, IDH mut, PFGFR, p53 status) was selected (in our case this was the k-means clustering). When the analyses were repeated by removing genes with extreme values (only 2 such genes), the results did not significantly change. All analyses were done in R version 3.6.1 and its libraries fpc (used for visualizations) [67] and cluster (used for PAM clustering) [68].

#### 4.10.2. Differentially Expressed Genes among Tissues and Glioblastoma Subtypes

We analyzed the differences in the expression of *CCL5* and *CCR5* among sample types; N-non-cancerous brain tissues; glioma I-II- low-grade gliomas; glioma III; GB-glioblastoma; GB rec- recurrent glioblastoma; GB cells; GSCs in the first analysis and between previously defined subtypes (mesenchymal—MES, proneural—PN, classical—CL subtype and finally the subtype combination—MIX) in the second analysis. To minimize the effect of genes with a low expression we first removed them from the analysis by replacing the Ct values > 40 as zero. We then assessed the overall similarity of genes/samples (sample types in the first analysis and subtypes in the second one) by hierarchical clustering and plotted the heatmaps for visual inspection of the results. The differential expression analyses were done using linear models and the empirical Bayes method to moderate the standard errors of the log of fold changes that were estimated with the linear model. In the first set of analyses we tested which genes differentially expressed when glioma or glioblastoma samples were compared with normal tissue when the gene expression in glioblastoma cells and GSCs was compared to normal astrocytes (NAS) when gene expression in glioblastoma samples was compared with N, glioma I and II, glioma III, and GB-rec and finally when gene expression in GSCs was compared with NAS and GB-cells. In the second experiment, samples were categorized according to their subtypes and the difference in gene expression between every pair of subtypes was tested. To enable an easier estimation of genes that were differentially expressed in two or more analyses we used Venn diagrams. All analyses were done in R version 3.6.1 and its libraries HTqPCR (used for data preprocessing and visualizations) [69], limma (used for differential expression calculations and Venn diagram visualizations) [70].

## Figures and Tables

**Table 1:** Immunohistochemical analyses of *CCL5* and *CCR5* expression in glioblastoma and non-cancerous tissues.

	CCR5			CCL5		
Tumor	Intensity	% Positive Cells	Localization	Intensity	% Positive Cells	Localization
Patient 2	+	1	c	−	0	/
Patient 5	−	0	/	+	1	c, e
Patient 8	+	1	c	++	3	c
Patient 9	−	0	/	−	0	/
NB 1	−	0	/	++	1	c
NB 2	+	2	c	++	2	c

IHC scoring was performed by a pathologist using a semi-quantitative grading system. This comprise the scoring immunostaining intensity: ++ moderate, + weak or no expression (−) and the abundance of stained cells percentage positive cells: 0; 1–33% = 1; 33–66% = 2; 66–100% = 3. The intracellular localization was evaluated as m = membrane, n = nuclear, c = cytoplasmic; e = extracellular, (/) = not determined.

**Table 2:** Clinical and histological parameters of glioblastoma patients.

Patients	Age (Years)	Gender	Survival * (Months)	Diagnosis	Necrosis **	Angiogenesis **	Karnofsky Score ***	Subtype (MES, PN, CL, MIX) ****	IDH *****
Patient 1	51	F	3	GB	yes	yes	90	ND	wt
Patient 2	54	M	10	GB	yes	yes	80	MIX	wt
Patient 3	61	M	8	GB	yes	yes	70	PN	wt
Patient 4	76	F	16	GB	no	yes	50	ND	n.a.
Patient 5	61	M	8	GB	no	yes	80	MIX	wt
Patient 8	51	M	6	GB	no	yes	100	CL	wt
Patient 9	67	M	9	GB	no	no	80	ND	wt
Patient 12	63	F	9	GB	no	no	90	CL	wt

* Survival: from the date of the first operation until death, GB—glioblastoma. ** Necrosis and angiogenesis were analyzed as »yes« or »no« by observation of the obtained glioblastoma tissue sample, before processing of the sample. *** Karnofsky score (at the time of the first operation) was determined by the clinician. Patient’s functional impairment; 80–100: normal activity, able to work, no special care needed; 50–70: unable to work, able to live at home, a varying amount of assistance needed; 0–40: unable to care for self, hospital care. **** Glioblastoma subtypes, mesenchymal (MES), proneural (PN) classical (CL), and MIX were determined based on the pattern of mRNA expression levels of selected genes, according to Behnan et al. (2017). n.a.: not available, ND: not determined. ***** IDH = Isocitrate dehydrogenase enzyme mutations were determined at the Pathology; wt-wild type, non-mutated.

**Table 3:** Immunocytochemical analyses of *CCL5* and *CCR5* expression in primary glioblastoma cells.

Cell Sample Name	CCR5 *	CCL5 *
Patient 1	−	+
Patient 2	+	+
Patient 3	−	−
Patient 5	+	++
Patient 8	++	+++
U373	−	−

* Immunocytochemical grades: +++ strong, ++ moderate, + weak, − no expression.

## Supplementary Materials

The following are available online at https://www.mdpi.com/1422-0067/21/12/4199/s1, Figure S1: Fluorescence immunocytochemical staining of *CCL5* and *CCR5* in bone-marrow derived mesenchymal stem cells (BM-MSC). Figure S2: Normal astrocytes do not express *CCL5* and *CCR5*. Figure S3: Cell viability of primary glioblastoma cells is not affected by *CCR5* inhibitor Maraviroc. Table S1: List of assays used for RT-qPCR analysis (Thermo Fisher Scientific, USA).
