# Supplementary material for: CCR5-Mediated Signaling is Involved in Invasion of Glioblastoma Cells in Its Microenvironment
Source: Int J Mol Sci. 2020 Jun 12;21(12):4199. doi: 10.3390/ijms21124199 (PMC7352708; doi:10.3390/ijms21124199)
Supplement: Supplementary file 1 [file ijms-21-04199-s001.pdf]

International Journal of *Molecular Sciences*

MDPI

Supplementary Materials

CCR5-mediated signaling is involved in invasion of glioblastoma cells in its microenvironment

Metka Novak[1], Miha Koprivnikar Krajnc[1], Barbara Hrastar[1], Barbara Breznik[1], Bernarda Majc[1,2], Mateja Mlinar[1], Ana Rotter[1], Andrej Porčnik[3], Jernej Mlakar[4], Katja Stare[5], Richard G. Pestell [6,*] and Tamara T Lah[1,7,*]

TABLES

Table S1: List of assays used for RT-qPCR analysis (Thermo Fisher Scientific, USA).

Probe name	Gene name	assay ID	assay type	Cat.number
**GAPDH**	*GAPDH*	Hs99999905_m1	FAM-MGB, S(250rxns)	4331182
**HPRT1**	*HPRT1*	Hs02800695_m1	FAM-MGB, XS(75rxns)	4453320
**CCL5**	*CCL5*	Hs00982282_m1	FAM-MGB, XS(75rxns)	4331182
**CCR5**	*CCR5*	Hs00152917_m1	FAM-MGB, XS(75rxns)	4331182

FIGURES

FIGURE S1

Here, we were interested, if MSCs alone, and when in glioblastoma microenvironment express CCL5 and CCR5. Therefore, we have immunolabelled bone marrow-derived MSCs in monocultures by CCL5 and CCR5 antibodies, and demonstrated high expression of both antigens in MSCs.

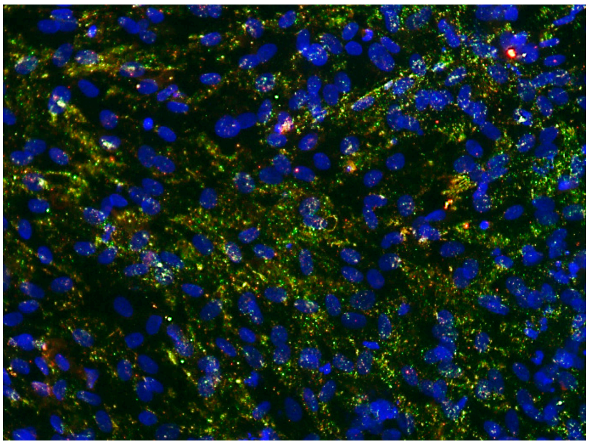

Figure S1. Fluorescence immunocytochemical staining of CCL5 and CCR5 in bone-marrow derived mesenchymal stem cells (BM-MSC). Fluorescence staining was performed as described in Materials and Methods, using antigen specific antibodies to detect CCL5 and CCR5 expressions in BM-MSC. Nuclei were stained with DAPI (blue), CCR5 with Alexa Fluor 488 (green) dye and CCL5 with Alexa Fluor 546 (red) dye. Coexpression of CCL5 and CCR5 appeared as yellow staining. Fluorescence microscope was used to analyse the cells at 20x magnification.

FIGURE S2

CCL5 and CCR5 are not expressed in normal astrocytes, which implicates for the CCL5-CCR5 axis importance in glioblastoma cells.

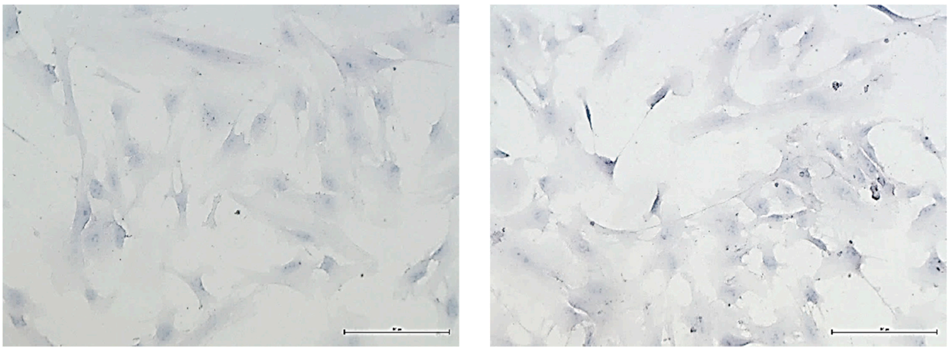

Figure S2. Normal astrocytes do not express CCL5 and CCR5. Immunocytochemical staining of CCL5 and CCR5 (brown) in normal astrocytes. Cell nuclei were counterstained by haematoxylin (blue). Scale bar represents 100 μm. Immunocytochemistry (IHC) analysses were performed using antibodies against CCR5 (ab65850, abcam) and CCL5-RANTES (ab189841, Abcam).

FIGURE S3

Glioblastoma cells' invasion was significantly enhanced after CCL5 stimulation, but was inhibited by maraviroc addition. Noteworthy, glioblastoma cells' viability was not affected by maraviroc even at high concentrations, showing functional significance of CCL5/CCR5 axis in glioblastoma invasion mechanism.

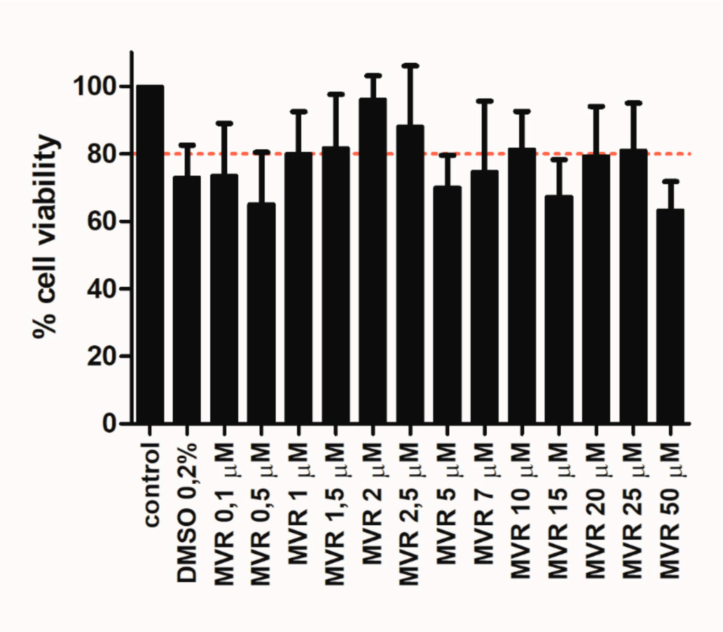

Figure S3. Cell viability of primary glioblastoma cells is not affected by CCR5 inhibitor Maraviroc. Cell viability of primary glioblastoma cells derived from patient Nb.2 (both CCR5 and CCL5 are expressed) is not affected by CCR5 inhibitor MVR (maraviroc). Cell viability was measured with MTT assay after 48h treatment. Each value represents the mean ± SD (n = 3). Briefly, cells were seeded into 96-well plates (8000 cells/ well) and grown overnight. Cells were treated with different concentrations of MVR (0.1-50 μM). Stock solutions of MVR were prepared in dimethyl sulfoxide (DMSO, Sigma-Aldrich). Control incubations contained the same amount of DMSO (0.2 %, v/v) as in all the treatment conditions. After 48 h, MTT was added. After 3 h incubation, the formazan crystals were dissolved in DMSO and the absorbance was measured as the change in optical density (ΔOD 570/690 nm) using microplate reader (Synergy™ HT, Bio-Tec Instruments Inc., USA). Cell viability was analysed using GraphPad Prism software.
